# Supplementary material for: Comparative chloroplast genomes and phylogenetic relationships of Aglaonema modestum and five variegated cultivars of Aglaonema
Source: PLoS One. 2022 Sep 2;17(9):e0274067. doi: 10.1371/journal.pone.0274067 (PMC9439221; doi:10.1371/journal.pone.0274067)
Supplement: S1 Fig — Genes shown inside the circle are transcribed clockwise, and those outside are transcribed counterclockwise. The gray arrowheads indicate the direction of the genes. Different genes are color coded. The innermost darker gray corresponds to the GC content, whereas the lighter gray corresponds to the AT content. The inner circle also indicates that the chloroplast genome contains a large single copy (LSC) region, a small single copy (SSC) region and two copies of the inverted repeat (IRA and IRB). (A) ‘Red Valentine’, (B) ‘Hong Yan’, (C) ‘Hong Jian’, (D) ‘Lady Valentine’, and (E) ‘Red Vein’. * in (D) and (E) indicates ycf68 and trnI-CAU only present in the IR regions of the chloroplast genomes of ‘Lady Valentine’ and ‘Red Vein’, respectively. (DOC) [file pone.0274067.s001.doc]

**
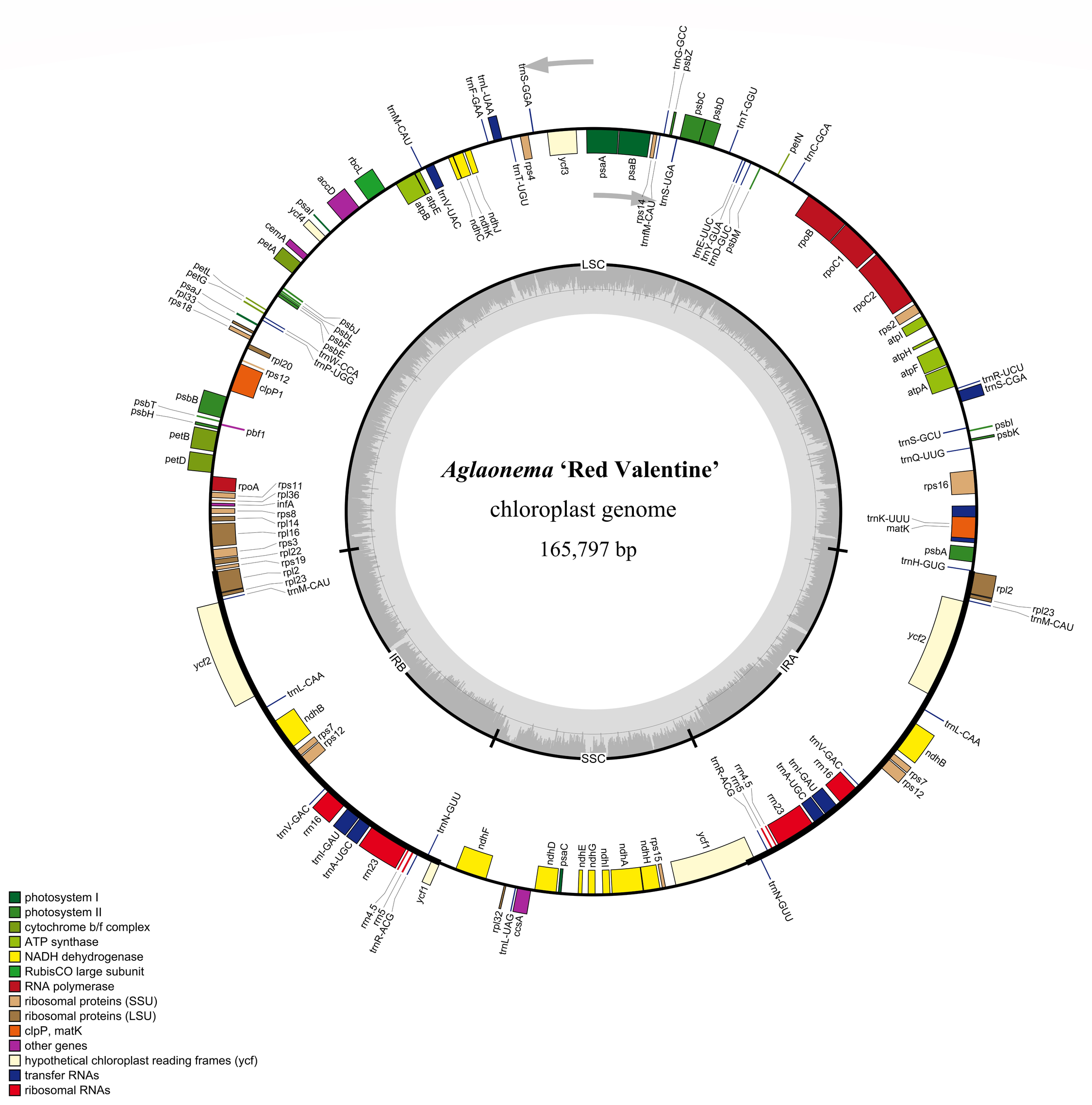
Fig S1.** Chloroplast genome maps of five variegated cultivars of *Aglaonema* in this study. Genes shown inside the circle are transcribed clockwise, and those outside are transcribed counterclockwise. The gray arrowheads indicate the direction of the genes. Different genes are color coded. The innermost darker gray corresponds to GC content, whereas the lighter gray corresponds to AT content. The inner circle also indicates that the chloroplast genome contains a large single copy region (LSC), a small single copy region (SSC) and two copies of the inverted repeat (IRA and IRB). (A) ‘Red Valentine’, (B) ‘Hong Yan’, (C) ‘Hong Jian’, (D) ‘Lady Valentine’, and (E) ‘Red Vein’. * in (D) and (E) indicates *ycf68* and *trnI-CAU* only present in the IR regiongs of chloroplast genomes of ‘Lady Valentine’ and ‘Red Vein’.

A


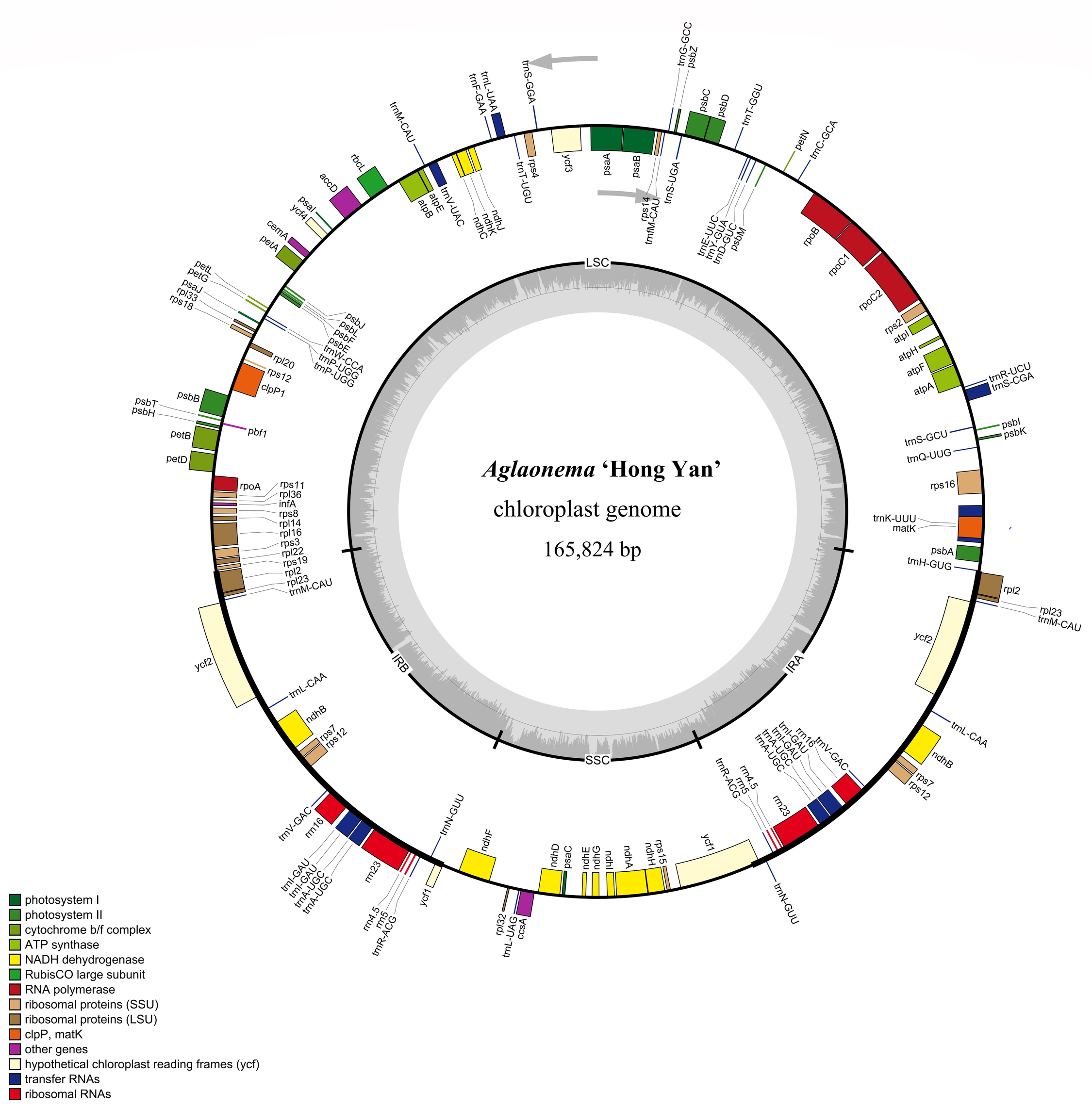


B

**Fig S1. continued.**


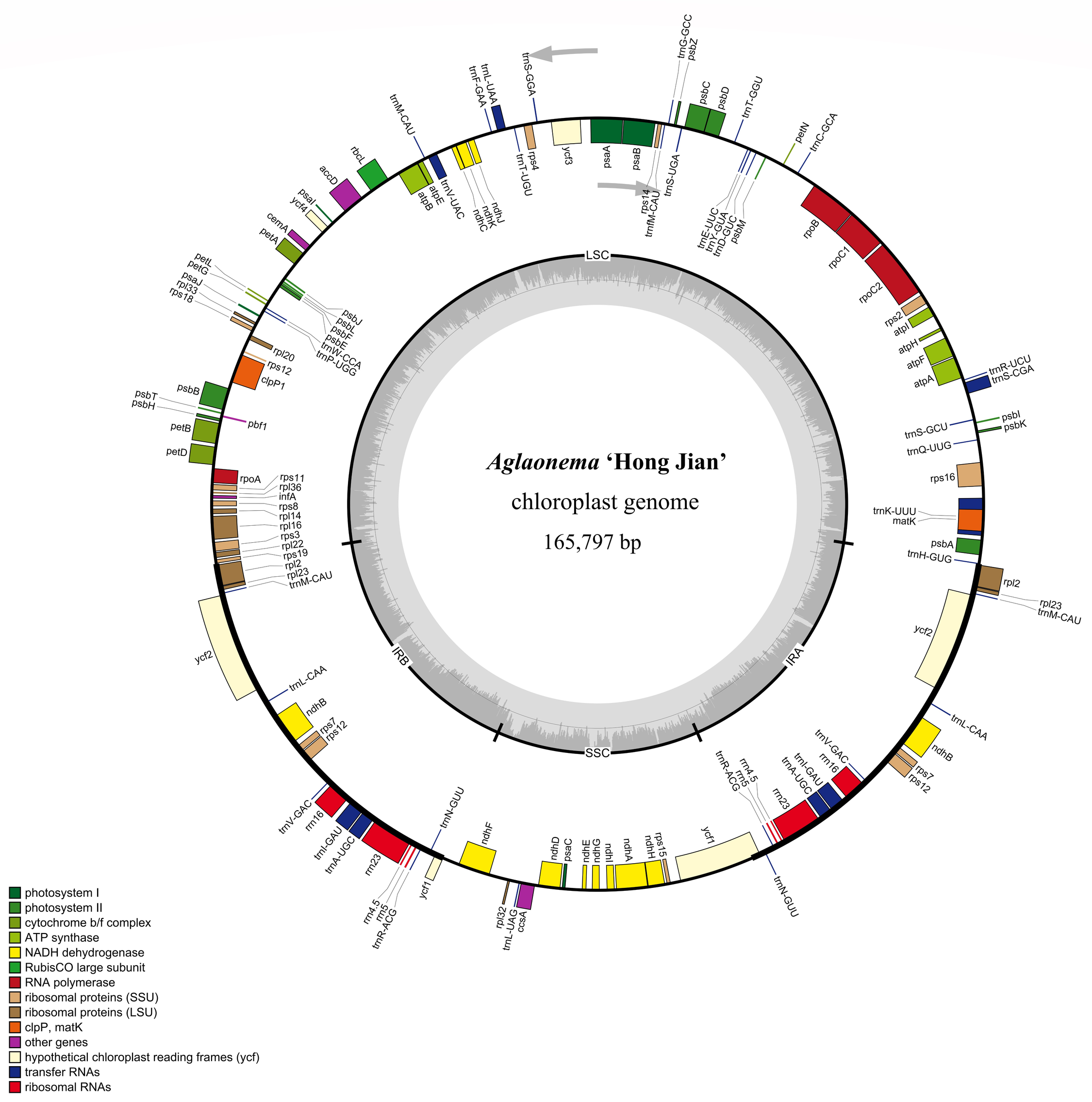


C

**Fig S1. continued.**

D

**
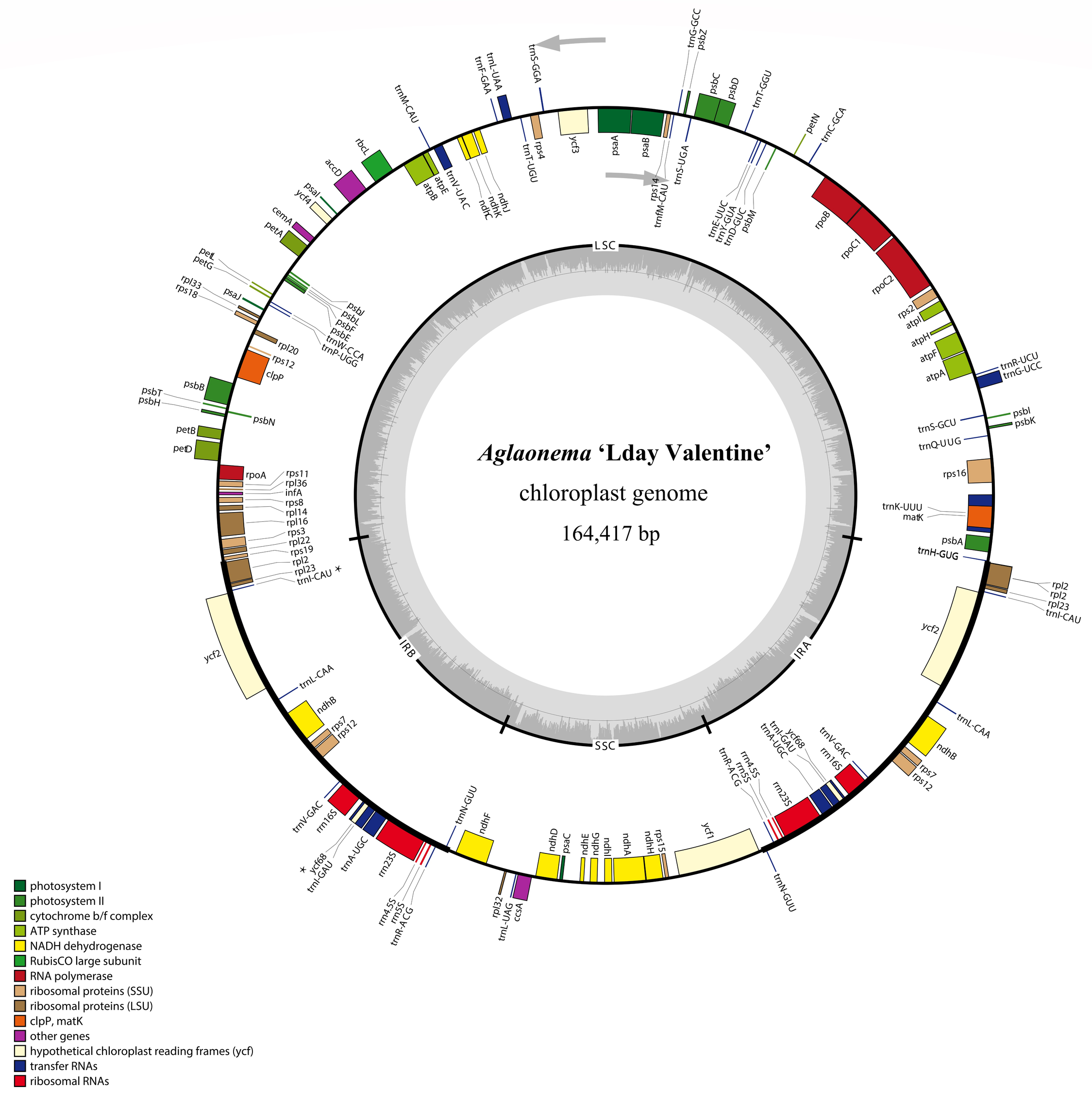
**

**Fig S1. continued.**

E

**
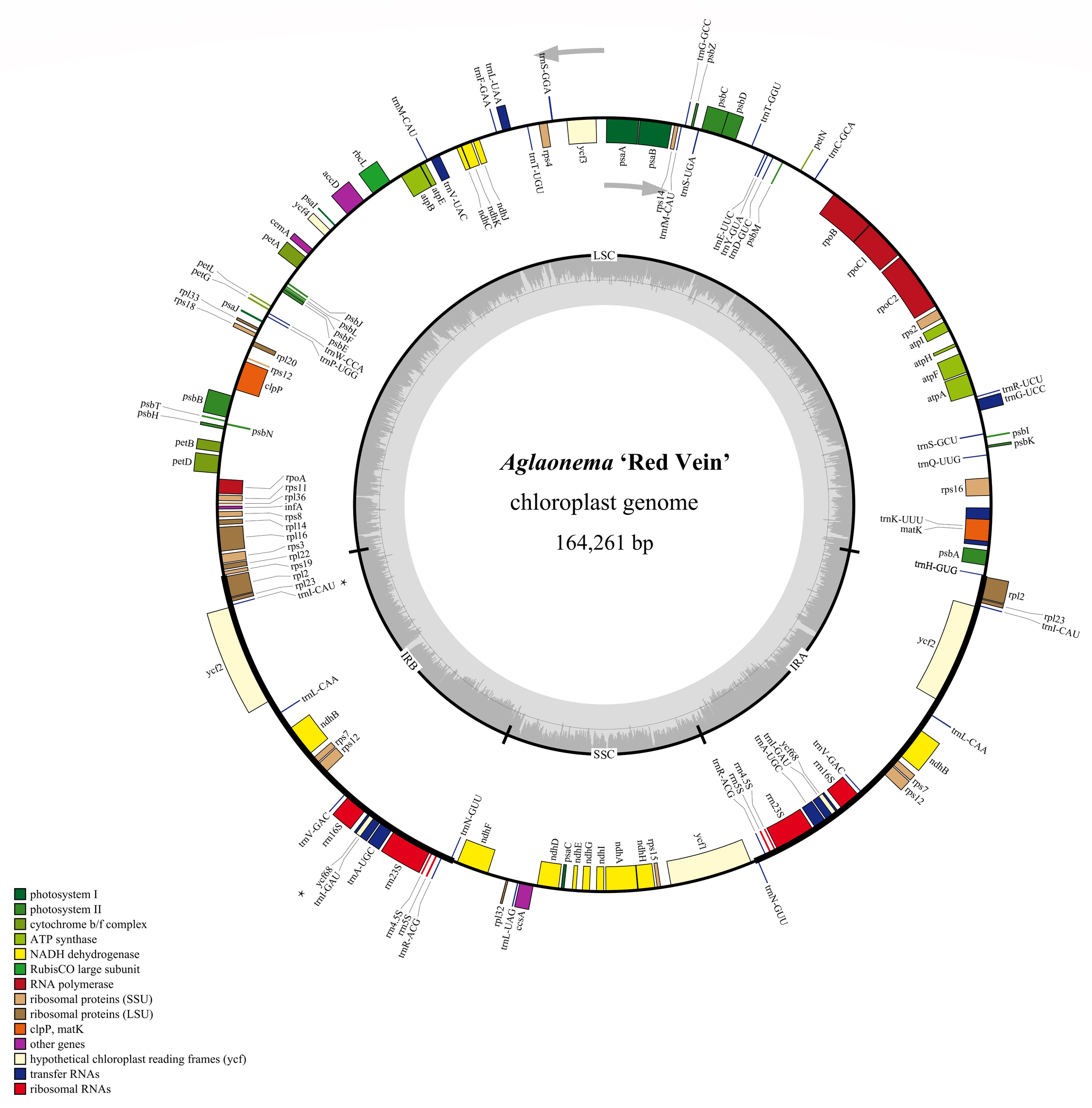
**

**Fig S1. continued.**
