# Supplementary material for: Comparative chloroplast genomes and phylogenetic relationships of Aglaonema modestum and five variegated cultivars of Aglaonema
Source: PLoS One. 2022 Sep 2;17(9):e0274067. doi: 10.1371/journal.pone.0274067 (PMC9439221; doi:10.1371/journal.pone.0274067)
Supplement: S2 Table — (DOCX) [file pone.0274067.s003.docx]

**Table S2** Features of six newly sequenced chloroplast genomes of *Aglaonema*.

| **Species/cultivars** | **Regions** | **Positions** | **Length(bp)** | **T/U(%)** | **C(%)** | **A(%)** | **G(%)** | **AT/U(%)** |
| --- | --- | --- | --- | --- | --- | --- | --- | --- |
| *A. modestum* | Genome |  | 165,626 | 32.56 | 18.22 | 31.61 | 17.61 | 64.17 |
|  | LSC |  | 91,269 | 33.82 | 17.38 | 32.2 | 16.6 | 66.02 |
|  | IRa |  | 26,732 | 28.89 | 20.16 | 29.49 | 21.46 | 58.38 |
|  | SSC |  | 20,893 | 35.69 | 15.25 | 35.2 | 13.87 | 70.88 |
|  | IRb |  | 26,732 | 29.49 | 21.46 | 28.89 | 20.16 | 58.38 |
|  | Protein coding genes |  | 80,010 | 31.38 | 17.6 | 30.93 | 20.09 | 62.31 |
|  |  | 1st position | 26,670 | 23.76 | 18.5 | 30.95 | 26.79 | 54.72 |
|  |  | 2nd position | 26,670 | 32.31 | 20.43 | 29.51 | 17.75 | 61.82 |
|  |  | 3rd position | 26,670 | 38.08 | 13.86 | 32.33 | 15.73 | 70.41 |
|  | tRNA |  | 2,803 | 25.12 | 23.72 | 21.83 | 29.33 | 46.95 |
|  | rRNA |  | 9,050 | 18.9 | 23.54 | 26.03 | 31.54 | 44.93 |
| *A.*‘Red valentine’ | Genome |  | 165,797 | 32.61 | 18.17 | 31.66 | 17.57 | 64.26 |
|  | LSC |  | 91,092 | 33.75 | 17.38 | 32.25 | 16.62 | 66 |
|  | IRa |  | 26,501 | 28.92 | 20.17 | 29.42 | 21.49 | 58.33 |
|  | SSC |  | 21,703 | 36.2 | 14.92 | 35.28 | 13.6 | 71.47 |
|  | IRb |  | 26,501 | 29.41 | 21.49 | 28.92 | 20.17 | 58.33 |
|  | Protein coding genes |  | 79,668 | 31.38 | 17.6 | 30.93 | 20.09 | 62.31 |
|  |  | 1st position | 26,556 | 23.76 | 18.5 | 30.9 | 26.83 | 54.66 |
|  |  | 2nd position | 26,556 | 32.34 | 20.38 | 29.54 | 17.74 | 61.87 |
|  |  | 3rd position | 26,556 | 38.04 | 13.92 | 32.35 | 15.69 | 70.39 |
|  | tRNA |  | 2,803 | 25.12 | 23.72 | 21.91 | 29.25 | 47.02 |
|  | rRNA |  | 9,050 | 18.94 | 23.49 | 26.03 | 31.54 | 44.97 |
| *A.* ‘Hong yan’ | Genome |  | 165,824 | 32.61 | 18.16 | 31.66 | 17.57 | 64.27 |
|  | LSC |  | 91,092 | 33.75 | 17.38 | 32.25 | 16.62 | 66 |
|  | IRa |  | 26,501 | 28.92 | 20.17 | 29.42 | 21.49 | 58.33 |
|  | SSC |  | 21,730 | 36.23 | 14.91 | 35.28 | 13.58 | 71.51 |
|  | IRb |  | 26,501 | 29.41 | 21.49 | 28.92 | 20.17 | 58.33 |
|  | Protein coding genes |  | 79,530 | 31.39 | 17.62 | 30.9 | 20.09 | 62.29 |
|  |  | 1st position | 26,510 | 23.78 | 18.52 | 30.85 | 26.85 | 54.63 |
|  |  | 2nd position | 26,510 | 32.35 | 20.4 | 29.51 | 17.74 | 61.86 |
|  |  | 3rd position | 26,510 | 38.06 | 13.94 | 32.33 | 15.67 | 70.39 |
|  | tRNA |  | 3,263 | 25.74 | 23.23 | 21.45 | 29.57 | 47.2 |
|  | rRNA |  | 9,050 | 18.94 | 23.49 | 26.03 | 31.54 | 44.97 |
| *A.* ‘Hong jian’ | Genome |  | 165,797 | 32.61 | 18.17 | 31.66 | 17.57 | 64.26 |
|  | LSC |  | 91,092 | 33.75 | 17.38 | 32.25 | 16.62 | 66 |
|  | IRa |  | 26,501 | 28.92 | 20.17 | 29.42 | 21.49 | 58.33 |
|  | SSC |  | 21,703 | 36.2 | 14.92 | 35.28 | 13.6 | 71.47 |
|  | IRb |  | 26,501 | 29.41 | 21.49 | 28.92 | 20.17 | 58.33 |
|  | Protein coding genes |  | 79,668 | 31.38 | 17.6 | 30.93 | 20.09 | 62.31 |
|  |  | 1st position | 26,556 | 23.76 | 18.5 | 30.9 | 26.83 | 54.66 |
|  |  | 2nd position | 26,556 | 32.34 | 20.38 | 29.54 | 17.74 | 61.87 |
|  |  | 3rd position | 26,556 | 38.04 | 13.92 | 32.35 | 15.69 | 70.39 |
|  | tRNA |  | 2,803 | 25.12 | 23.72 | 21.91 | 29.25 | 47.02 |
|  | rRNA |  | 9,050 | 18.94 | 23.49 | 26.03 | 31.54 | 44.97 |

**Table S2** continued.

| **Species/cultivars** | **Regions** | **Positions** | **Length(bp)** | **T/U(%)** | **C(%)** | **A(%)** | **G(%)** | **AT/U(%)** |
| --- | --- | --- | --- | --- | --- | --- | --- | --- |
| *A. ‘*Lady valentine’ | Genome |  | 164,417 | 32.52 | 18.24 | 31.61 | 17.63 | 64.13 |
|  | LSC |  | 91,135 | 33.77 | 17.38 | 32.25 | 16.61 | 66.02 |
|  | IRa |  | 25,788 | 28.66 | 20.31 | 29.37 | 21.67 | 58.02 |
|  | SSC |  | 21,706 | 35.64 | 15.31 | 35.11 | 13.94 | 70.75 |
|  | IRb |  | 25,788 | 28.66 | 20.3 | 29.36 | 21.67 | 58.02 |
|  | Protein coding genes |  | 77,400 | 31.26 | 17.61 | 31.03 | 20.11 | 62.28 |
|  |  | 1st position | 25,800 | 23.74 | 18.55 | 30.94 | 26.77 | 54.68 |
|  |  | 2nd position | 25,800 | 32.14 | 20.37 | 29.7 | 17.79 | 61.84 |
|  |  | 3rd position | 25,800 | 37.89 | 13.91 | 32.44 | 15.76 | 70.33 |
|  | tRNA |  | 2,798 | 24.87 | 23.52 | 22.12 | 29.49 | 47 |
|  | rRNA |  | 9,050 | 18.94 | 23.49 | 26.03 | 31.54 | 44.97 |
| *A. ‘*Red vein’ | Genome |  | 164,261 | 32.47 | 18.25 | 31.63 | 17.65 | 64.09 |
|  | LSC |  | 91,769 | 33.75 | 17.33 | 32.34 | 16.57 | 66.1 |
|  | IRa |  | 25,838 | 28.71 | 20.2 | 29.51 | 21.58 | 58.22 |
|  | SSC |  | 20,816 | 35.12 | 15.78 | 34.71 | 14.39 | 69.83 |
|  | IRb |  | 25,838 | 28.71 | 20.19 | 29.53 | 21.57 | 58.23 |
|  | Protein coding genes |  | 79,623 | 31.27 | 17.72 | 30.83 | 20.18 | 62.1 |
|  |  | 1st position | 26,541 | 23.78 | 18.6 | 30.74 | 26.88 | 54.52 |
|  |  | 2nd position | 26,541 | 32.18 | 20.5 | 29.46 | 17.86 | 61.64 |
|  |  | 3rd position | 26,541 | 37.85 | 14.07 | 32.28 | 15.8 | 70.13 |
|  | tRNA |  | 2,798 | 24.87 | 23.52 | 22.12 | 29.49 | 47 |
|  | rRNA |  | 9,050 | 18.94 | 23.49 | 26.03 | 31.54 | 44.97 |
