# Supplementary material for: Comparative chloroplast genomes and phylogenetic relationships of Aglaonema modestum and five variegated cultivars of Aglaonema
Source: PLoS One. 2022 Sep 2;17(9):e0274067. doi: 10.1371/journal.pone.0274067 (PMC9439221; doi:10.1371/journal.pone.0274067)
Supplement: S3 Table — (DOCX) [file pone.0274067.s004.docx]

**Table S3 Genes with introns in the six newly sequenced chloroplast genomes of genus *Aglaonema*.**

| **Species/ cultivar** | **Gene** | **Location** | **Exon Ⅰ(bp)** | **Intron Ⅰ(bp)** | **Exon Ⅱ(bp)** | **Intron Ⅱ(bp)** | **Exon Ⅲ(bp)** |
| --- | --- | --- | --- | --- | --- | --- | --- |
| *A. modestum* | *trnA-UGC* | IR | 38 | 799 | 35 |  |  |
|  | *trnS-CGA* | LSC | 31 | 713 | 60 |  |  |
|  | *trnI-GAU* | IR | 32 | 938 | 40 |  |  |
|  | *trnK-UUU* | LSC | 36 | 2556 | 38 |  |  |
|  | *trnL-UAA* | LSC | 37 | 503 | 50 |  |  |
|  | *trnV-UAC* | LSC | 31 | 609 | 31 |  |  |
|  | *rps12** | LSC/IR | 114 |  | 232 | 546 | 26 |
|  | *rps16* | LSC | 195 | 1402 | 42 |  |  |
|  | *rpl2* | IR | 431 | 664 | 403 |  |  |
|  | *rpl16* | LSC | 399 | 1206 | 9 |  |  |
|  | *petB* | LSC | 6 | 765 | 642 |  |  |
|  | *petD* | LSC | 8 | 744 | 475 |  |  |
|  | *atpF* | LSC | 401 | 839 | 145 |  |  |
|  | *ndhA* | SSC | 539 | 1102 | 553 |  |  |
|  | *ndhB* | IR | 756 | 685 | 777 |  |  |
|  | *rpoC1* | LSC | 1629 | 740 | 423 |  |  |
|  | *clpP* | LSC | 246 | 630 | 292 | 810 | 71 |
|  | *ycf3* | LSC | 153 | 781 | 228 | 727 | 132 |
| *A.*‘Red valentine’ | *trnA-UGC* | IR | 38 | 799 | 35 |  |  |
|  | *trnS-CGA* | LSC | 31 | 713 | 60 |  |  |
|  | *trnI-GAU* | IR | 32 | 947 | 40 |  |  |
|  | *trnK-UUU* | LSC | 36 | 2538 | 38 |  |  |
|  | *trnL-UAA* | LSC | 37 | 518 | 50 |  |  |
|  | *trnV-UAC* | LSC | 31 | 630 | 31 |  |  |
|  | *rps12** | LSC/IR | 114 | - | 232 | 546 | 26 |
|  | *rps16* | LSC | 197 | 1423 | 40 |  |  |
|  | *rpl2* | IR | 431 | 664 | 403 |  |  |
|  | *rpl16* | LSC | 399 | 1221 | 9 |  |  |
|  | *petB* | LSC | 6 | 786 | 642 |  |  |
|  | *petD* | LSC | 8 | 763 | 475 |  |  |
|  | *atpF* | LSC | 401 | 850 | 145 |  |  |
|  | *ndhA* | SSC | 539 | 1100 | 553 |  |  |
|  | *ndhB* | IR | 756 | 686 | 777 |  |  |
|  | *rpoC1* | LSC | 1620 | 742 | 432 |  |  |
|  | *clpP* | LSC | 246 | 638 | 292 | 788 | 71 |
|  | *ycf3* | LSC | 153 | 797 | 230 | 742 | 124 |

*The *rps12* gene is divided into 5’-*rps12* in the LSC region and 3’-*rps12* in the IR region.

**Table S3 continued.**

| **Species/ cultivar** | **Gene** | **Location** | **Exon Ⅰ(bp)** | **Intron Ⅰ(bp)** | **Exon Ⅱ(bp)** | **Intron Ⅱ(bp)** | **Exon Ⅲ(bp)** |
| --- | --- | --- | --- | --- | --- | --- | --- |
| *A.* ‘Hong yan’ | *trnA-UGC* | IR | 38 | 799 | 35 |  |  |
|  | *trnS-CGA* | LSC | 31 | 713 | 60 |  |  |
|  | *trnI-GAU* | IR | 32 | 947 | 40 |  |  |
|  | *trnK-UUU* | LSC | 36 | 2538 | 38 |  |  |
|  | *trnL-UAA* | LSC | 37 | 518 | 50 |  |  |
|  | *trnV-UAC* | LSC | 31 | 630 | 31 |  |  |
|  | *rps12** | LSC/IR | 114 |  | 232 | 546 | 26 |
|  | *rps16* | LSC | 197 | 1423 | 40 |  |  |
|  | *rpl2* | IR | 431 | 664 | 403 |  |  |
|  | *rpl16* | LSC | 399 | 1221 | 9 |  |  |
|  | *petB* | LSC | 6 | 786 | 642 |  |  |
|  | *petD* | LSC | 8 | 763 | 475 |  |  |
|  | *atpF* | LSC | 401 | 850 | 145 |  |  |
|  | *ndhA* | SSC | 539 | 1100 | 553 |  |  |
|  | *ndhB* | IR | 756 | 686 | 777 |  |  |
|  | *rpoC1* | LSC | 1620 | 742 | 432 |  |  |
|  | *clpP* | LSC | 246 | 638 | 292 | 788 | 71 |
|  | *ycf3* | LSC | 153 | 797 | 230 | 742 | 124 |
| *A.* ‘Hong jian’ | *trnA-UGC* | IR | 38 | 799 | 35 |  |  |
|  | *trnS-CGA* | LSC | 31 | 713 | 60 |  |  |
|  | *trnI-GAU* | IR | 32 | 947 | 40 |  |  |
|  | *trnK-UUU* | LSC | 36 | 2538 | 38 |  |  |
|  | *trnL-UAA* | LSC | 37 | 516 | 50 |  |  |
|  | *trnV-UAC* | LSC | 31 | 630 | 31 |  |  |
|  | *rps12** | LSC/IR | 114 | - | 232 | 546 | 26 |
|  | *rps16* | LSC | 197 | 1423 | 40 |  |  |
|  | *rpl2* | IR | 431 | 664 | 403 |  |  |
|  | *rpl16* | LSC | 399 | 1221 | 9 |  |  |
|  | *petB* | LSC | 6 | 786 | 642 |  |  |
|  | *petD* | LSC | 8 | 763 | 475 |  |  |
|  | *atpF* | LSC | 401 | 850 | 145 |  |  |
|  | *ndhA* | SSC | 539 | 1100 | 553 |  |  |
|  | *ndhB* | IR | 756 | 686 | 777 |  |  |
|  | *rpoC1* | LSC | 1620 | 742 | 432 |  |  |
|  | *clpP* | LSC | 246 | 638 | 292 | 788 | 71 |
|  | *ycf3* | LSC | 153 | 797 | 230 | 742 | 124 |

**Table S3 continued.**

| **Species/ cultivar** | **Gene** | **Location** | **Exon Ⅰ(bp)** | **Intron Ⅰ(bp)** | **Exon Ⅱ(bp)** | **Intron Ⅱ(bp)** | **Exon Ⅲ(bp)** |
| --- | --- | --- | --- | --- | --- | --- | --- |
| *A. ‘*Lday valentine’ | *trnA-UGC* | IR | 38 | 799 | 35 |  |  |
|  | *trnG-UCC* | LSC | 24 | 731 | 48 |  |  |
|  | *trnI-GAU* | IR | 42 | 942 | 35 |  |  |
|  | *trnK-UUU* | LSC | 44 | 2535 | 37 |  |  |
|  | *trnL-UAA* | LSC | 37 | 518 | 50 |  |  |
|  | *trnV-UAC* | LSC | 37 | 586 | 39 |  |  |
|  | *rps12** | LSC/IR | 114 |  | 237 | 546 | 27 |
|  | *rps16* | LSC | 196 | 1423 | 41 |  |  |
|  | *rpl2* | IR | 431 | 664 | 391 |  |  |
|  | *rpl16* | LSC | 399 | 1221 | 9 |  |  |
|  | *petB* | LSC | 6 | 53 | 642 |  |  |
|  | *petD* | LSC | 7 | 763 | 476 |  |  |
|  | *atpF* | LSC | 401 | 850 | 145 |  |  |
|  | *ndhA* | SSC | 539 | 1100 | 553 |  |  |
|  | *ndhB* | IR | 756 | 686 | 777 |  |  |
|  | *rpoC1* | LSC | 1623 | 739 | 453 |  |  |
|  | *clpP* | LSC | 246 | 638 | 292 | 788 | 71 |
|  | *ycf3* | LSC | 153 | 797 | 229 | 742 | 125 |
|  | *ycf68* | IR | 42 | 31 | 303 |  |  |
| *A. ‘*Red vein’ | *trnA-UGC* | IR | 38 | 799 | 35 |  |  |
|  | *trnG-UCC* | LSC | 24 | 731 | 48 |  |  |
|  | *trnI-GAU* | IR | 42 | 942 | 35 |  |  |
|  | *trnK-UUU* | LSC | 44 | 2540 | 37 |  |  |
|  | *trnL-UAA* | LSC | 37 | 516 | 50 |  |  |
|  | *trnV-UAC* | LSC | 37 | 589 | 39 |  |  |
|  | *rps12** | LSC/IR | 114 | - | 231 | 546 | 27 |
|  | *rps16* | LSC | 196 | 1001 | 41 |  |  |
|  | *rpl2* | IR | 431 | 664 | 391 |  |  |
|  | *rpl16* | LSC | 399 | 1269 | 9 |  |  |
|  | *petB* | LSC | 6 | 53 | 642 |  |  |
|  | *petD* | LSC | 7 | 743 | 476 |  |  |
|  | *atpF* | LSC | 401 | 832 | 145 |  |  |
|  | *ndhA* | SSC | 539 | 1103 | 553 |  |  |
|  | *ndhB* | IR | 756 | 686 | 777 |  |  |
|  | *rpoC1* | LSC | 1623 | 739 | 453 |  |  |
|  | *clpP* | LSC | 246 | 643 | 292 | 811 | 71 |
|  | *ycf3* | LSC | 153 | 786 | 229 | 737 | 125 |
|  | *ycf68* | IR | 42 | 31 | 303 |  |  |
