# Supplementary material for: Comparative chloroplast genomes and phylogenetic relationships of Aglaonema modestum and five variegated cultivars of Aglaonema
Source: PLoS One. 2022 Sep 2;17(9):e0274067. doi: 10.1371/journal.pone.0274067 (PMC9439221; doi:10.1371/journal.pone.0274067)
Supplement: Attachment — Submitted filename: response to editor and reviewers.19.August.doc [file pone.0274067.s013.doc]

Journal Requirements:

When submitting your revision, we need you to address these additional requirements.

1. Please ensure that your manuscript meets PLOS ONE's style requirements, including those for file naming. The PLOS ONE style templates can be found at
   https://journals.plos.org/plosone/s/file?id=wjVg/PLOSOne_formatting_sample_main_body.pdf and 
   <https://journals.plos.org/plosone/s/file?id=ba62/PLOSOne_formatting_sample_title_authors_affiliations.pdf>

Reponse: We revised the style requirements one by one.

2. We note that the grant information you provided in the ‘Funding Information’ and ‘Financial Disclosure’ sections do not match. When you resubmit, please ensure that you provide the correct grant numbers for the awards you received for your study in the ‘Funding Information’ section.

Reponse: We checked the ‘Funding Information’ section and found the correct grant numbers for the awards. We added “Guangdong Province Modern Agriculture Industry Technical System-Flower Innovation Team Construction Project (2021KJ121)”, because if the manuscript is accepted, the publication fee is from this project (2021KJ121).

When we returned the manuscript, we found no ‘Financial Disclosure’ section in the questions. Hence, we wrote ‘**Funding**’ information behind ‘**Data Availability Statement**’.

**Funding:** This research was financially supported by the Guangdong Basic and Applied Basic Foundation Project (No. 2021A1515010893), Special Financial Fund of Foshan-High-level Guangdong Agricultural Science and Technology Demonstration City Project (2022), and Guangdong Province Modern Agriculture Industry Technical System-Flower Innovation Team Construction Project (2021KJ121). The funders had no role in study design, data collection and analysis, decision to publish, or preparation of the manuscript.

3. Thank you for stating the following in your Competing Interests section:  
    "No." 
Please complete your Competing Interests on the online submission form to state any Competing Interests. If you have no competing interests, please state "The authors have declared that no competing interests exist.", as detailed online in our guide for authors at http://journals.plos.org/plosone/s/submit-now 
 This information should be included in your cover letter; we will change the online submission form on your behalf.

Reponse: The authors have declared that no competing interests exist.

4. In your Data Availability statement, you have not specified where the minimal data set underlying the results described in your manuscript can be found. PLOS defines a study's minimal data set as the underlying data used to reach the conclusions drawn in the manuscript and any additional data required to replicate the reported study findings in their entirety. All PLOS journals require that the minimal data set be made fully available. For more information about our data policy, please see http://journals.plos.org/plosone/s/data-availability.

"Upon re-submitting your revised manuscript, please upload your study’s minimal underlying data set as either Supporting Information files or to a stable, public repository and include the relevant URLs, DOIs, or accession numbers within your revised cover letter. For a list of acceptable repositories, please see http://journals.plos.org/plosone/s/data-availability#loc-recommended-repositories. Any potentially identifying patient information must be fully anonymized.

Important: If there are ethical or legal restrictions to sharing your data publicly, please explain these restrictions in detail. Please see our guidelines for more information on what we consider unacceptable restrictions to publicly sharing data: http://journals.plos.org/plosone/s/data-availability#loc-unacceptable-data-access-restrictions. Note that it is not acceptable for the authors to be the sole named individuals responsible for ensuring data access.

We will update your Data Availability statement to reflect the information you provide in your cover letter.

Reponse: The six newly assembled chloroplast genomes in this study have been deposited in GenBank with accession numbers MK262737, MK262738, OK094434, OK094435, OK094436, and OK094427.
Additional Editor Comments:

In this study, chloroplast genomes were generated and used to elucidate the phylogenetic relationships of cultivated Aglaonema in South China. The genomes are valuable resources for Aglaonema. Substantial improvement and edition in language may be needed, with attention to many long sentences and redundant content. Here are a few examples and the authors should go through the entire paper.
Title:
Replace “foliar variegation” (a noun) with “variegated” (an adjective) before “cultivar” (a noun). They refer to the same botanical character. Remove the family name at the end, which is unnecessary.
Likewise, replace all “foliar variegation” with “variegated” in the entire paper if the two words are used as an adjective before “cultivar”.
Reponse: Yes, we agreed and revised.
Abstract:
Line 14-16: Reorganize the sentence and remove the redundant word Aglaonema. All words in cultivar names should be capitalized as they are specific nouns.
Reponse: Yes, we agreed and revised.

Line 20-23, this long sentence contains many grammatical errors. For example, “that” should be ”these”. “were significant different” should be “were significantly different” or “had significant differences”. Should it be “distinctive” (typical) or “distinct” (different)?
Reponse: line 20, “that” should not be ”these”. We revised as following: but the IR-SSC boundary regions were significantly different, and ‘Red Vein’ had a distinct long repeat number and type frequency.

We agreed with the revisions of line 21-23. “were significant different” should be “were significantly different” . “distinctive” should be “distinct”.

Line 22, remove “A. “ before ‘Red vein’ and all cultivar names, unnecessary and awkward.
Reponse: Yes, we agreed and revised.

Line 28, How are they “positive selection”? are they just spontaneous mutations accumulated in the genomes?
Reponse: Positive selection was found for *rpl2*, *rps2*, *rps3*, *ycf1* and *ycf2* based on the analyses of Ka/Ks ratios among 16 Araceae chloroplast genomes.
Introduction:
Line 45: “has started since 1930s and soon” should change to “started in 1930s and”
Reponse: Yes, we agreed and revised.

Line 47: remove the hyphen in “sports-selected”
Reponse: Yes, we agreed and revised.

Line 50-51, “Aglaonema species and cultivars identifications” may be better as “identification of Aglaonema species and cultivars”
……
Reponse: Yes, we agreed and revised.

M&M
Line 82-84, move this section elsewhere following the journal’s instruction.
Reponse: Yes, we agreed and revised.

Line 85, should be “materials”
Reponse: Yes, we agreed and revised.

Line 86-87, remove all “A.” before all cultivars and capitalize all cultivar names
……
Reponse: Yes, we agreed and revised. We improved the English writing quality of the manuscript with help from American Journal Experts (AJE).

[Note: HTML markup is below. Please do not edit.]
Reviewers' comments:
Reviewer's Responses to Questions

**Comments to the Author**
1. Is the manuscript technically sound, and do the data support the conclusions?
The manuscript must describe a technically sound piece of scientific research with data that supports the conclusions. Experiments must have been conducted rigorously, with appropriate controls, replication, and sample sizes. The conclusions must be drawn appropriately based on the data presented.

Reviewer #1: Partly

Reviewer #2: Partly

2. Has the statistical analysis been performed appropriately and rigorously?

Reviewer #1: N/A

Reviewer #2: Yes

3. Have the authors made all data underlying the findings in their manuscript fully available?

The [PLOS Data policy](http://www.plosone.org/static/policies.action#sharing) requires authors to make all data underlying the findings described in their manuscript fully available without restriction, with rare exception (please refer to the Data Availability Statement in the manuscript PDF file). The data should be provided as part of the manuscript or its supporting information, or deposited to a public repository. For example, in addition to summary statistics, the data points behind means, medians and variance measures should be available. If there are restrictions on publicly sharing data—e.g. participant privacy or use of data from a third party—those must be specified.

Reviewer #1: Yes

Reviewer #2: Yes

4. Is the manuscript presented in an intelligible fashion and written in standard English?

PLOS ONE does not copyedit accepted manuscripts, so the language in submitted articles must be clear, correct, and unambiguous. Any typographical or grammatical errors should be corrected at revision, so please note any specific errors here.

Reviewer #1: Yes

Reviewer #2: No

Reponse: We have improved the English writing quality of the manuscript with help from American Journal Experts (AJE).

5. Review Comments to the Author

Please use the space provided to explain your answers to the questions above. You may also include additional comments for the author, including concerns about dual publication, research ethics, or publication ethics. (Please upload your review as an attachment if it exceeds 20,000 characters)

Reviewer #1: The authors reported chloroplast genomes of Aglaonema modestum and five Aglaonema foliar variegation cultivars. The results enriched chloroplast genome data of the genus, thus is of some value. But I don't think it is suitable for publication is its present form, before the authors make a substantial revision.
1. Separate Discussion section from the Result section, and strengthen it to elucidate the findings.

Reponse:Yes, we agreed. We separated ‘Results and Discussion’ into two sections: ‘Result’ and ‘Discusssion’. Details of ‘Discusssion’ can be found in the revised manuscript.

2. Introduce clearly the relationship between Aglaonema modestum and five Aglaonema foliar variegation cultivars selected.

Reponse: Yes, we introduced clearly the leaf morphological charateristics of *A. modestum* and five variegated cultivars in the section ‘Plant Material’ as following:

*A. modestum* is erect with a dark green stem and leaves, which is not variegated (Fig. 1A). Both ‘Red Valentine’ and ‘Lady Valentine’ are variegated, with green and red along the midrib, along veins and throughout the leaf blade (Fig. 1B-1C). The leaves of ‘Hong Jian’ are narrow, oblong, dark pink to dark red, accounting for approximately 75% to 90% of the leaf area, and the shape of the apex of the leaves is strongly acute, with green and dark yellow green blotches at the margin and throughout the leaf blade (Fig. 1D). The leaves of ‘Hong Yan’ are ovate-cordate, dark red, accounting for approximately 95% of the leaf area, and the shape of the apex of the leaves is obtuse to moderately acute, with green blotches at the leaf margin (Fig. 1E). The leaves of ‘Red Vein’ are dark green with pink to red along the midrib and veins (Fig. 1F).

3. I can not agree with the conclusion "Phylogenetic analysis indicated that A. modestum was close to A. costatum, and that the five foliar variegation cultivars could be clearly classified." To draw such a conclusion, more samples of each species/cultivar are needed.

Reponse: Thank you very much for this suggestion. We revised the "Phylogenetic analyses” section and revised the conclusion as following:

In this study, two phylogenetic trees were constructed by ML method using whole chloroplast genomes and protein coding genes, respectively (Fig 10A-10B). *A. americanus* was classified in family Acoraceae [32], which was used as the outgroup. The 7 subfamilies within Araceae, including Aroideae, Lasioideae, Lemnoideae, Monsteroideae, Orontioideae, Pothoideae, and Zamioculcadoideae, could be stongly identified by the whole chloroplast genomes with bootstrap values 89-100% (Fig 10A). In contrast, the phylogenetic tree constructed by protein coding genes showed moderate (bootstrap value = 84%) to strong identifications (bootstrap value = 100%) (Fig 10B). Within subfamily Aroideae, *Aglaonema* was strongly supported as monophyletic (bootstrap values = 100%) (Fig 10A-10B). *Aglaonema* was seen as either sister to *Anchomanes* (bootstrap value = 100%), and *Aglaonema* + *Anchomanes* and *Zantedeschia* were strongly supported as sister genera (bootstrap value = 88%) (Fig 10A), or formed a poorly-supported clade with *Stylochaeton* (bootstrap value = 17%) that was sister to genera *Anchomanes* + *Zantedeschia* (bootstrap value = 100%) (Fig 10B). Within genus *Aglaonema*, ‘Hong Jian’, ‘Red Valentine’, ‘Hong Yan’, and ‘Lady Valentine’ were clustered together with moderate to strong support (bootstrap values = 80-100%), and ‘Red Vein’ was sister to these four variegated cultivars with strong support (bootstrap value = 100%) (Fig 10A). *A. costatum* was sister to these five variegated cultivars with strong support (bootstrap value = 99%). *A. modestum* was sister to the clade of “*A. costatum* *+* five variegated cultivars” with strong support (bootstrap value = 100%) based on complete chloroplat genomes (Fig 10A). These seven *Aglaonema* species and cultivars showed similar topologies based on protein coding genes, but ‘Hong Jian’ and ‘Red Valentine’ were poorly supported in the ML analysis (bootstrap value = 0) (Fig 10B). The other five *Aglaonema* species and cultivars were strongly supported in ML analysis based on protein coding genes (bootstrap values = 89-100%) (Fig 10B).

Reviewer #2: In this manuscript, authors compared chloroplast genome on one green species and five foliar variegation cultivars of Aglaonema. Authors have done detail comparison and characterization of the six genomes. The study might be interesting for publication in PLOS ONE. However, the manuscript does have several issues which I think need to be addressed.
Major
1. Authors point out that it is difficulty to identify Aglaonema species and cultivars based on leaf morphology. It seems there is no clear answer to that question. Phylogenetic analysis just shows the genetic relationship among Aglaonema species and cultivars. It could be very useful if authors provide molecular markers to identify or separate Aglaonema species and cultivars, especially for ‘two mutations Hong yan’ and ‘Hong jian’, as well as ‘Red valentine.

Reponse: Yes, we provided molecular markers to identify or separate among *A. modestum*, *A. costatum*, ‘Red valentine’, ‘Lady Valentine’, and ‘Red Vein’ (Table 3, S10 Table, and S11 Table).

It is difficult to identify two cultivars ‘Red valentine’ and ‘Lady Valentine’ by their leaf morphological charateristics. In this study, it was worth noting that 14 SNPs and 1 insertion were found between these two cultivars genomes. These 14 SNPs were all located in *psaA* (S10 Table), and 1 insertion was found in *rps12* (S11 Table). Hence, *psaA* and *rps12* sequences can be used to differentiate these two cultivars. However, two mutations ‘Hong yan’ and ‘Hong jian’, as well as ‘Red valentine, had no SNPs/indels, which indicated that the chloroplast genomes had not undergone recombination in two tissue-culture induced mutations of ‘Red Valentine’. The two mutations, ‘Hong Jian’ and ‘Hong Yan’ may be induced by nuclear gene variation and complex biological processes. The molecular regulation mechanisms of leaf shape and leaf colour variations of ‘Hong Jian’ and ‘Hong Yan’, need further investigation.

2. There is limited discussion, and most are presented results in ‘Results and Discussion’. For example, in line 157, “Interestingly, both A. ‘Red valentine’ and A. ‘Hong jian’ were with same genome size, which was 158 bp smaller than that of A. ‘Hong yan’. However, there is no explanation or discussion why authors think this is an interesting result. I suggested to separate ‘Results and Discussion’ into two sections: Result’, ‘Discussion’.

Reponse: Yes, we agreed. We separated ‘Results and Discussion’ into two sections: ‘Result’ and ‘Discusssion’. Details of ‘Discusssion’ can be found in the revised manuscript.
Minor
3. line 52-56, the background information of the plant materials (Fig.1) should be moved to the section ‘Plant Material’.

Reponse: Yes, we agreed. We moved Fig.1 to the section ‘Plant Material’.

4. Fig2, except genome sizes, there is no other information, such as genes, GC content for six sequenced chloroplast genomes of genus Aglaonema.

Reponse: We revised Fig.2 to display chloroplast genome map of *A. modestum* and added the other five sequenced chloroplast genomes maps in S1 Fig. The genes, GC content and other genome information were showed in Table 1, S1 Table, and S2 Table.

5. Fig3 B, it should be interesting to show a cluster for the six sequenced chloroplast genomes. Heatmap shows they are all the same and no difference.

Reponse: Yes, we agreed. As we draw the Fig 3B using RSCU values with two decimal data, it looked no difference. We revised Fig 3B using all four decimal data as following, which existed differences.

**Fig 3.** (B) Heat map analysis for codon distribution of all protein coding genes in six newly sequenced chloroplast genomes. * indicates stop codons.

6. PLOS authors have the option to publish the peer review history of their article ([what does this mean?](https://journals.plos.org/plosone/s/editorial-and-peer-review-process#loc-peer-review-history)). If published, this will include your full peer review and any attached files.

If you choose “no”, your identity will remain anonymous but your review may still be made public.

**Do you want your identity to be public for this peer review?** For information about this choice, including consent withdrawal, please see our [Privacy Policy](https://www.plos.org/privacy-policy).

Reviewer #1: No

Reviewer #2: No

[NOTE: If reviewer comments were submitted as an attachment file, they will be attached to this email and accessible via the submission site. Please log into your account, locate the manuscript record, and check for the action link "View Attachments". If this link does not appear, there are no attachment files.]

While revising your submission, please upload your figure files to the Preflight Analysis and Conversion Engine (PACE) digital diagnostic tool, <https://pacev2.apexcovantage.com/>. PACE helps ensure that figures meet PLOS requirements. To use PACE, you must first register as a user. Registration is free. Then, login and navigate to the UPLOAD tab, where you will find detailed instructions on how to use the tool. If you encounter any issues or have any questions when using PACE, please email PLOS at [figures@plos.org](mailto:figures@plos.org). Please note that Supporting Information files do not need this step.

Reponse: We revised the figures by PACE on line.
